# The Importance of Real-World Validation of Machine Learning Systems in Wearable Exercise Biofeedback Platforms: A Case Study

**DOI:** 10.3390/s21072346

**Published:** 2021-03-27

**Authors:** Rob Argent, Antonio Bevilacqua, Alison Keogh, Ailish Daly, Brian Caulfield

**Affiliations:** 1 Insight Centre for Data Analytics, University College Dublin, D04 V1W8 Dublin, Ireland; antonio.bevilacqua@insight-centre.org (A.B.); alison.keogh@insight-centre.org (A.K.); b.caulfield@ucd.ie (B.C.); 2 School of Public Health, Physiotherapy and Sport Science, University College Dublin, D04 V1W8 Dublin, Ireland; 3 School of Computer Science, University College Dublin, D04 V1W8 Dublin, Ireland; 4 Beacon Hospital, D18 AK68 Dublin, Ireland; ailish.daly@beaconhospital.ie

**Keywords:** biofeedback, biomedical technology, exercise therapy, machine learning, human factors, wearables, inertial measurement unit

## Abstract

Machine learning models are being utilized to provide wearable sensor-based exercise biofeedback to patients undertaking physical therapy. However, most systems are validated at a technical level using lab-based cross validation approaches. These results do not necessarily reflect the performance levels that patients and clinicians can expect in the real-world environment. This study aimed to conduct a thorough evaluation of an example wearable exercise biofeedback system from laboratory testing through to clinical validation in the target setting, illustrating the importance of context when validating such systems. Each of the various components of the system were evaluated independently, and then in combination as the system is designed to be deployed. The results show a reduction in overall system accuracy between lab-based cross validation (>94%), testing on healthy participants (*n* = 10) in the target setting (>75%), through to test data collected from the clinical cohort (*n* = 11) (>59%). This study illustrates that the reliance on lab-based validation approaches may be misleading key stakeholders in the inertial sensor-based exercise biofeedback sector, makes recommendations for clinicians, developers and researchers, and discusses factors that may influence system performance at each stage of evaluation.

## 1. Introduction

Within physical rehabilitation, remotely collating and aggregating data from patients has been suggested to have numerous benefits in terms of cost, clinical outcome and patient satisfaction [1,2]. Exercise biofeedback systems use a sensing platform to capture and interpret data to offer the user meaningful information about their performance [3]. Many systems utilise one or more inertial measurement units (IMUs) to acquire biomechanical data [4,5,6,7,8]. Some systems perform simple data processing tasks such as repetition counting, whilst others use more complex supervised machine learning (ML) models to offer greater granularity of feedback to the user such as joint angle measurement, repetition segmentation, or exercise technique biofeedback [9,10,11].

**Figure 1:**
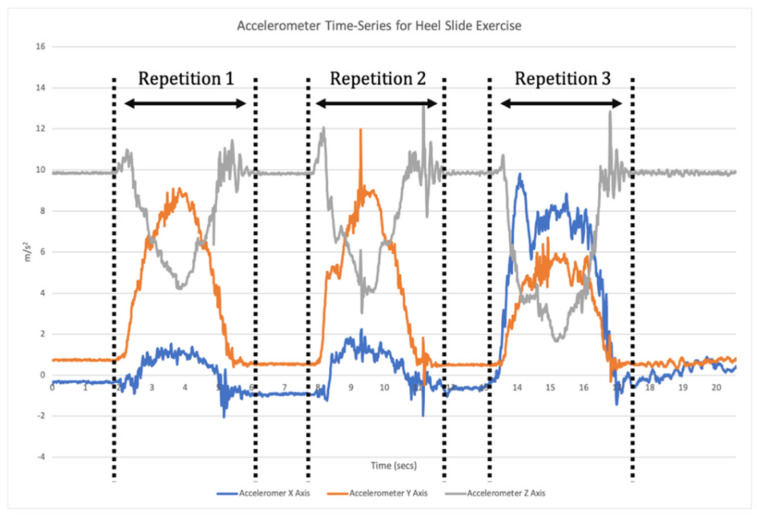
An example of a correctly segmented time-series of triaxial accelerometer data.

**Figure 2:**
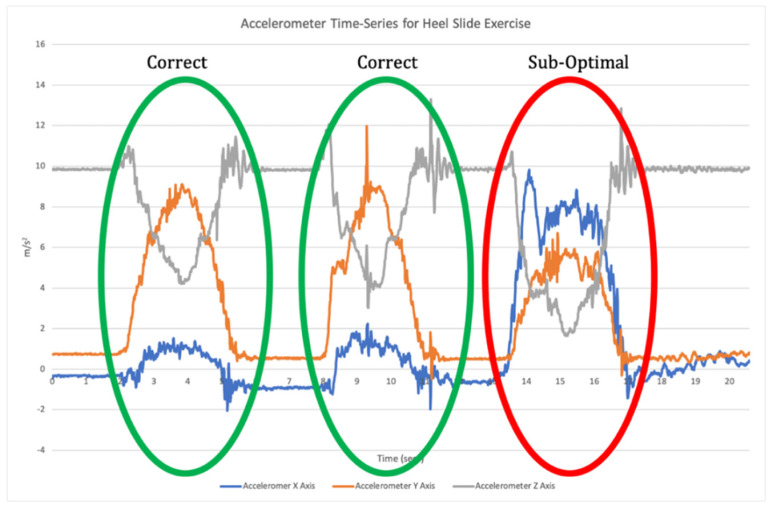
An example of classification of a time-series of triaxial accelerometer data with a sub-optimal repetition highlighted in red.

The application of ML spans a variety of biomechanical contexts, with models developed to predict the effect of an intervention, perform activity recognition, predict disease progression or classify abnormal movement [12]. Supervised learning is one of the main categories of ML and involves training a model which best maps input features to labelled outputs. This requires the developed algorithms to be provided with annotated training data, and the features analysed. The model is then trained using these data and the algorithms are tested with new unlabelled data to identify its accuracy [9]. For example, supervised ML algorithms can be used with data collected from an IMU to classify exercise performance and technique, whereby the output of the model can give a binary prediction as to whether an exercise in a sequence (or set) was performed correctly or not [10,13,14]. This requires a two-step data analysis process. Firstly, when a time-series of sensor data is recorded the signal needs to be broken down into each individual repetition of the exercise (segmentation; Figure 1). Segmentation goes beyond merely counting repetitions, but also isolates each individual repetition for the subsequent classification phase [11]. Once individual repetitions have been segmented, classification is undertaken where the input data for each repetition are compared to the labelled training data to assess which label they match most closely to (Figure 2). 

It is common practice to test a number of different ML algorithms for early validation, with the best performing algorithm being used within the final model [15,16]; however, there are suggestions that the common cross-validation methods for evaluating ML models analysing IMU data do not provide a realistic reflection of system performance [17]. 

The World Health Organisation has published guidelines for the evaluation of digital health interventions which include monitoring the functionality of a system, with functionality defined as *“the degree to which a product or system provides functions that meet stated and implied needs when used under specific conditions”* [18]. In the context of exercise biofeedback systems, we can assume that the ‘specific conditions’ relate to a real-world application that involves the patient performing exercise and receiving feedback outside a clinical or laboratory setting. However, there is a lack of research investigating the real-world validity of IMU based biofeedback systems with only one previous real-world validation of an exercise classification model identified in the literature [19]. Thus, there is a clear need to assess these components in real-world environments in order to optimise their effectiveness. A framework for segmentation model validation has recently been proposed to assess segmentation accuracy using a staged approach, with testing in laboratory, pre-clinical and clinical settings [20]. However, this does not consider the overall performance of an exercise biofeedback system incorporating classification models or continue to demonstrate an evaluation of a system in each of these settings. 

Therefore, the aim of this study was to conduct a thorough validation of an example IMU-based exercise biofeedback systems with testing in the laboratory through to clinical participants in the target-use conditions. Additionally, three objectives were identified; (1) to identify the accuracy of the classification models using the lab-based cross validation approach, (2) to investigate the performance of the segmentation and classification models independently with newly collected test data from a healthy and a clinical population, and (3) to evaluate overall biofeedback system performance with test data that have been algorithmically segmented and classified.

## 2. Materials and Methods

**Figure 3:**
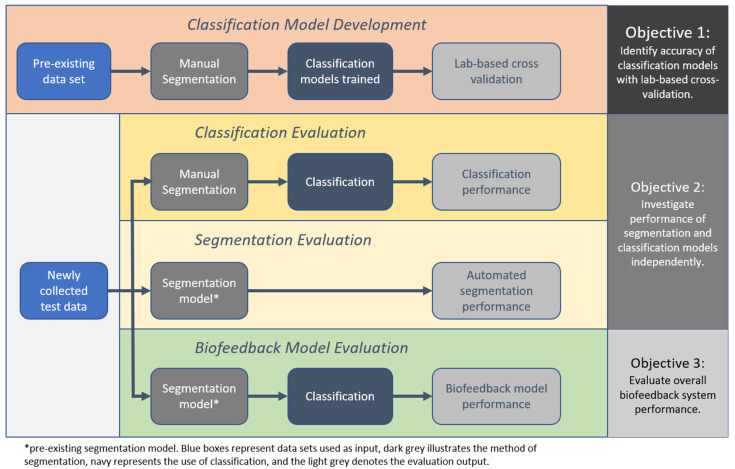
Steps of evaluation for the various machine learning components of the exercise biofeedback system.

The method of investigation was broken into four key phases: (1) the development of the classification models and lab-based evaluation, (2) evaluation of the classification models using manually segmented test data, (3) evaluation of the segmentation model, and (4) overall biofeedback model performance evaluation, combining the segmentation and classification models (Figure 3). 

### 2.1. Classification Model Development

#### 2.1.1. Training Data

A previously collected labelled data set was used to train the classification models [21]. This data set contained IMU data from clinical participants undergoing lower limb rehabilitation collected in a supervised setting. Participants performed the exercises both correctly, or with naturally occurring deviations, with the error labels chosen based on a Delphi survey identifying the commonly occurring deviations in these exercises [21]. A description of each of the exercises, and the error assessed is described in Table 1. These exercises were selected due to their widespread use in rehabilitation programmes following orthopaedic interventions such as knee arthroplasty [22,23]. Table 2 illustrates the composition of the training data, providing a breakdown of the number of participants, exercise sets, and balance of correct and sub-optimal performance of each exercise.

#### 2.1.2. Classification Model Design

To build the classification models, each IMU data set was manually segmented to isolate each individual repetition. A set of 352 features were then extracted for each repetition which were derived from nine different signal vectors, namely the acceleration and angular velocity in the x, y and z axes, plus the magnitude, pitch and roll [11]. For each of these vectors, two different groups of features which are commonly adopted for classification of IMU data [24,25]. 

Static features in the time domain (*n* = 14): mean, median, standard deviation, variance, range, kurtosis, skewness, maximum, minimum, positive mean, negative man, sum of absolute differences, 1st quartile, and 3rd quartile. 

Dynamic features in the frequency domain (*n* = 25): energy, energy ratio, energy average, harmonic ratio, energy entropy, and the first 20 coefficients of the signal Fourier transformation.

Additionally, the Pearson correlation coefficient between pitch and roll vectors is included in the feature set. These features were then used to train a number of classification algorithms [26], the selection of which were determined by previous exploratory work assessing for the most suitable algorithms in a similar data set [21]:

i. logistic regression
ii. support vector machine (SVM) trained with the sequential minimal optimisation algorithm (SMO) technique
iii. adaptive boosting
iv. random forest
v. J48 decision tree

Leave-one-subject-out cross-validation (LOSOCV), seen as the most appropriate cross-validation approach when considering an entirely new user [8,12], was then completed to assess the performance of each algorithm. The algorithm demonstrating the best accuracy per exercise was selected for use in the ML classification component of the biofeedback system and testing with newly collected data. 

#### 2.1.3. Classification Cross Validation

In line with similar work and as recommended, performance for the classification algorithms was measured using accuracy, sensitivity, and specificity metrics [12,13,21]. Accuracy (Equation (1)) is the number of correctly classified repetitions divided by the total number of repetitions, this is calculated by the sum of the number of true positives (TP) and true negatives (TN) divided by the sum of the true positives, false positives (FP), true negatives and false negatives (FN). Sensitivity (Equation (2)) refers to the effectiveness of the classifier to identify a desired positive label, in this case a correctly performed repetition, whilst specificity (Equation (3)) describes the ability of the model to detect a negative label—a sub-optimal performance of the exercise.

(1) $$\mathrm{Classification}\;\mathrm{Accuracy}=\frac{\mathrm{TP}+\mathrm{TN}}{\mathrm{TP}+\mathrm{FP}+\mathrm{TN}+\mathrm{FN}}$$

(2) $$\mathrm{Sensitivity}=\frac{\mathrm{TP}}{\mathrm{TP}+\mathrm{FN}}$$

(3) $$\mathrm{Specificity}=\frac{\mathrm{TN}}{\mathrm{TN}+\mathrm{FP}}$$

Equations (1)–(3) to calculate the accuracy, sensitivity and specificity of the classification models, respectively.

### 2.2. Test Data Collection

#### 2.2.1. Example IMU-Based Biofeedback System

**Figure 4:**
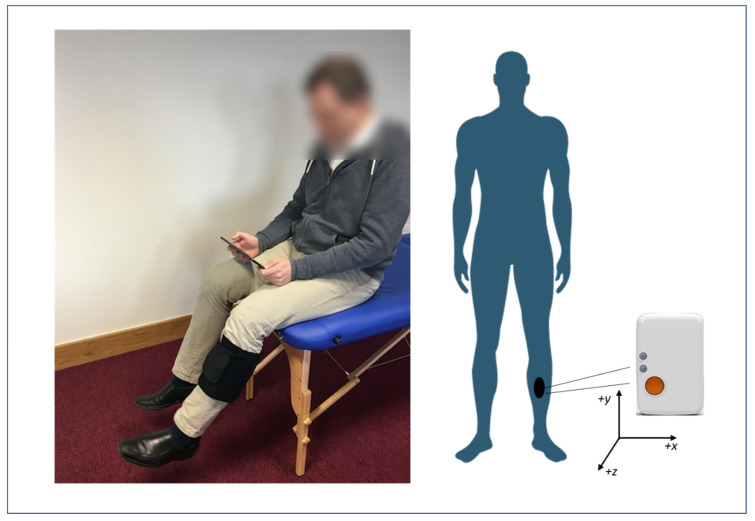
Illustration of IMU placement, orientation and user setup. Figure taken from Argent et al. (2019) [6].

A prototype biofeedback system for lower limb rehabilitation comprising of a single IMU (Shimmer, Dublin, Ireland) [27] and a custom-built Android tablet application was used to collect the test data for this study. The Shimmer3 IMU was configured to sample at 102.4 Hz and utilised a low-noise accelerometer (±2 g) and tri-axial gyroscope (500°/s). All units were calibrated according to the manufacturer instructions prior to testing using the Shimmer 9DOF Calibration Application v1.0 (Shimmer, Dublin, Ireland), and paired via Bluetooth to a corresponding tablet. The IMU was placed at the midpoint of the anterior aspect of the shin in a custom-made neoprene sleeve as illustrated in Figure 4. Further details of the system can be found in Argent et al. [6].

#### 2.2.2. Healthy Participants

In order to collect real-world test data, 10 participants (six female and four male, mean age = 66 years (range 57–91) were recruited from the general population. Participants were selected based on the similar age demographic to those of knee replacement patients [28]. They were required to be over 55 years of age and be capable of performing the four rehabilitation exercises. Participants were excluded if they had a history of lower limb musculoskeletal injury in the past six months, orthopaedic surgery in the past year, or previous bilateral knee replacement surgery.

#### 2.2.3. Clinical Participants

A sample of 11 participants (six male, five female, mean age = 62 years (range 49–71) were recruited for the clinical test data. Participants were recruited from a single private hospital in Dublin, Ireland, having recently undergone total or uni-compartmental knee replacement. The study received ethical approval from the Beacon Hospital Research Ethics Committee (BEA0065), and written informed consent was obtained from all participants prior to commencing the study.

#### 2.2.4. Experimental Procedure

Data were collected from participants in their own home using the example biofeedback system. Participants were provided with an explanation of each exercise and allowed the opportunity to practice three repetitions with the supervision of a Chartered Physiotherapist. The IMU was configured, calibrated, and placed on the anterior shin, and video was recorded from the trunk down for each exercise. Fifteen repetitions of each exercise were completed with the raw IMU data saved to the tablet device. Healthy participants completed a set of all exercises on one occasion, while clinical participants completed three supervised sessions, each one week apart, all participants completed the exercises to the best of their ability. Video data were then reviewed and labelled by two Chartered Physiotherapists and where there was discrepancy, a discussion took place between the Physiotherapists until agreement was reached, with each repetition labelled as correctly or sub-optimally performed.

### 2.3. Classification Evaluation

In order to determine the accuracy of each component, the first task was to assess the classification in isolation to the segmentation model. Each exercise file from the newly collected test set was manually segmented using a combination of physical boundaries and template boundaries [29], with labelled coordinates identifying each individual repetition within the time-series. These repetitions were then run through the classification model offline (not through the biofeedback application) to remove any software programming or computational load issues, and the accuracy, sensitivity and specificity of the classifiers were then calculated based on this manual segmentation. 

### 2.4. Segmentation Evaluation

A pre-existing segmentation model was deployed within the prototype system [15]. This utilises a template matching algorithm to firstly derive the periods of rest within the set of exercises. Periods of rest are then clustered to provide a reference point for each period, and pairs of consecutive reference points are tested against the template matching algorithm, with the template being the expected signal for a repetition. If the algorithm returns a positive result, where the signal matches, the pair of reference points are considered as start and end coordinates for a repetition. 

**Figure 5:**
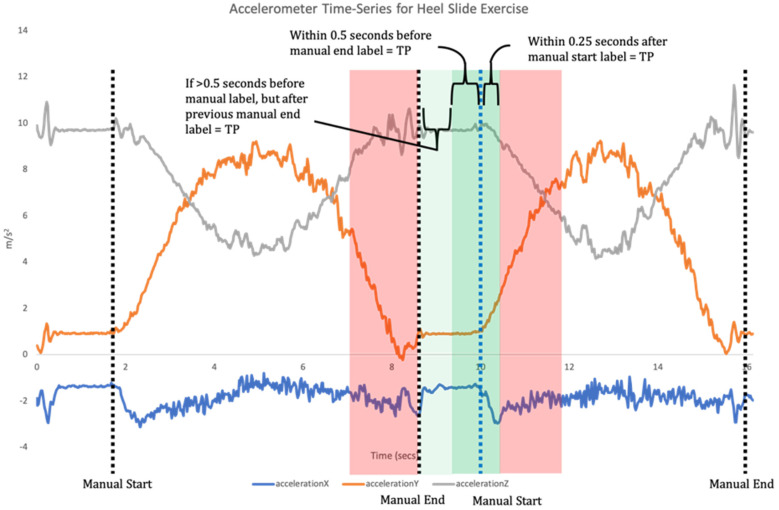
Illustration of threshold for segmentation for points identified as the start of a repetition. The manual annotation for reference is highlighted in blue and the area for TP in green.

For the prototype system to function effectively, the exercise files need to be segmented automatically, therefore the same test set was run offline through the segmentation model. The segmentation model performance was then assessed using temporal tolerance [29], where a TP is identified if the coordinates occur within a range (±*t*_err_) of the manually annotated label. The identification of a point from the model that is not manually identified in the ±*t*_err_ region is a FP error, and a FN error is recorded if a point was not found by the model in the ±*t*_err_ region of a manually annotated point. There is variance within the literature for the range of ±*t*_err_ [30,31,32]; however, for the purposes of this study, an asymmetrical ±*t*_err_ threshold was used. A threshold for points at the start of a repetition was set to 0.5 s before the manually annotated point, and 0.25 s after the same manually annotated point. However, if the predicted point was greater than 0.5 s before the annotation point, but after the end point for the previous repetition, this was also deemed acceptable, as there was no overlap (Figure 5).

**Figure 6:**
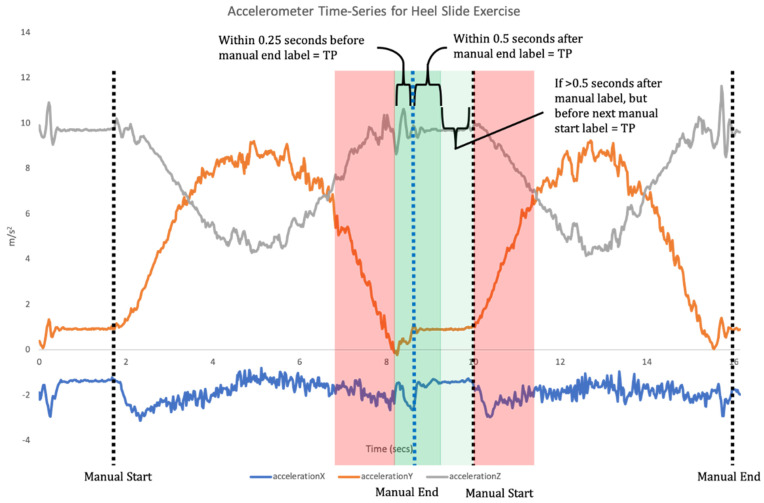
Illustration of threshold for segmentation for points identified as the end of a repetition. The manual annotation for reference is highlighted in blue and area for TP in green.

The opposite was used for the predicted points at the end of a repetition, where the threshold was 0.25 s before to 0.5 s after the manually annotated point, with a point greater than 0.5 s after the corresponding manual point but before the manually selected point for the start of the next repetition also being identified as a TP (Figure 6). 

Once the temporal tolerance process was used to assess the segmentation model point selection, the precision (Equation (4)), recall (Equation (5)) and accuracy (Equation (6)) were calculated [29].

(4) $$\mathrm{Precision}=\frac{\mathrm{TP}}{\mathrm{TP}+\mathrm{FP}}$$

(5) $$\mathrm{Recall}=\frac{\mathrm{TP}}{\mathrm{TP}+\mathrm{FN}}$$

(6) $$\mathrm{Recall}=\frac{\mathrm{TP}}{\mathrm{TP}+\mathrm{FN}}$$

Equations (4)–(6) were used to calculate the precision, recall and accuracy of the segmentation model, respectively. 

### 2.5. Biofeedback Model Evaluation

Finally, the classification accuracy was based on the segmentation algorithm outputs rather than manual labelling. This provides the true real-world classification accuracy when all components of the system are incorporated, as would be the case for the end-user. When determining the technical functionality of the models, and in line with previous research [33], classification accuracy was considered as ‘excellent’ when greater than 90%, ‘good’ when 80–89%, ‘moderate’ when 60–79%, and ‘poor’ when less than 59%.

## 3. Results

### 3.1. Lab-Based Cross-Validation

The results of the LOSOCV of binary classification for the best performing algorithm per exercise are presented in Table 3 with these models being used in the example exercise biofeedback system. The full results for all algorithms can be found in Supplementary File 1.

### 3.2. Test Data Characteristics

Table 4 outlines the composition of the test sets collected in this study. All participants in the healthy data set completed every repetition of each exercise with correct technique. Eleven clinical participants were recruited for this study; however, the data for one of these participants were compromised due to technical issues for three of the four exercises and were therefore discarded. 

### 3.3. Classification Performance

To evaluate the classification models in isolation to the segmentation algorithm, the test sets were manually segmented and run through the best performing classification models as outlined in Table 3. The mean classification accuracy along with the 95% confidence interval (CI) is illustrated in Table 5. In some cases, participants performed every repetition of the exercises correctly, with no sub-optimal examples (Table 4), therefore it was not possible to determine the specificity of the classification models for these exercises. 

As illustrated in Table 5, the mean classification accuracy of all four exercises when the segments were manually annotated was greater than 84% for healthy participants, with the system correctly classifying all repetitions in the heel slide (HS) and seated active knee extension (SAKE) exercises. In the clinical cohort, with the exception of the HS exercise which demonstrated excellent accuracy (mean = 98.99%), the mean classification accuracy ranged between 58.49–66.01% with IRQ demonstrating ‘poor’ performance (mean accuracy = 58.49%), and both SLR and SAKE offering ‘moderate’ performance when manually segmented. 

### 3.4. Segmentation Performance

**Figure 7:**
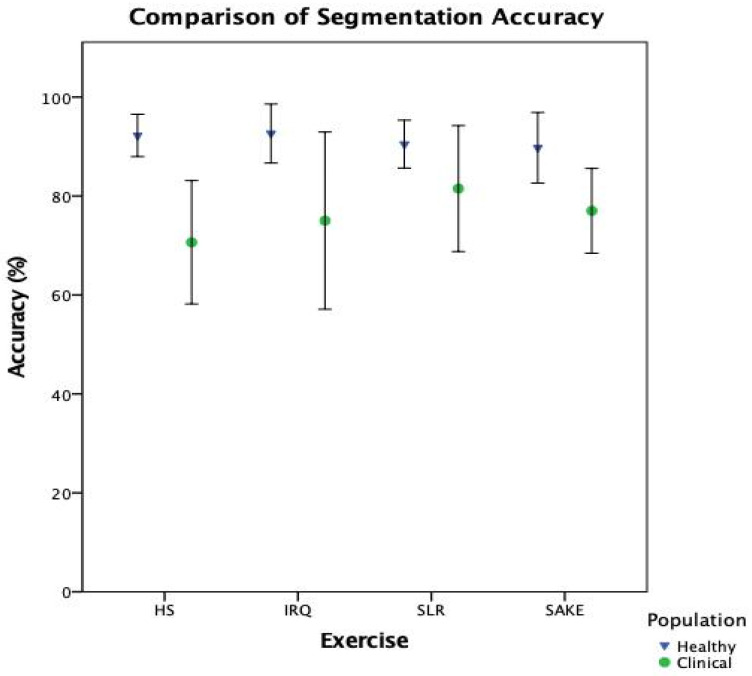
Comparison of segmentation accuracy between healthy and clinical test data.

The asymmetrical threshold was used to calculate the segmentation accuracy. Table 6 illustrates the mean segmentation performance along with the 95% CI, with average accuracy in the healthy cohort greater than 89% across all four exercises when compared to the manually annotated coordinates. In the clinical cohort, mean segmentation accuracy across the four exercises ranged from 70.64% to 81.50%. These results show a reduction in segmentation performance compared to testing with healthy data (Figure 7). There is an average reduction in segmentation accuracy across the four exercises of 15.24% ranging from SLR reducing by 8.98%, to HS demonstrating a 21.60% reduction in segmentation accuracy.

### 3.5. Biofeedback Model Performance

**Figure 8:**
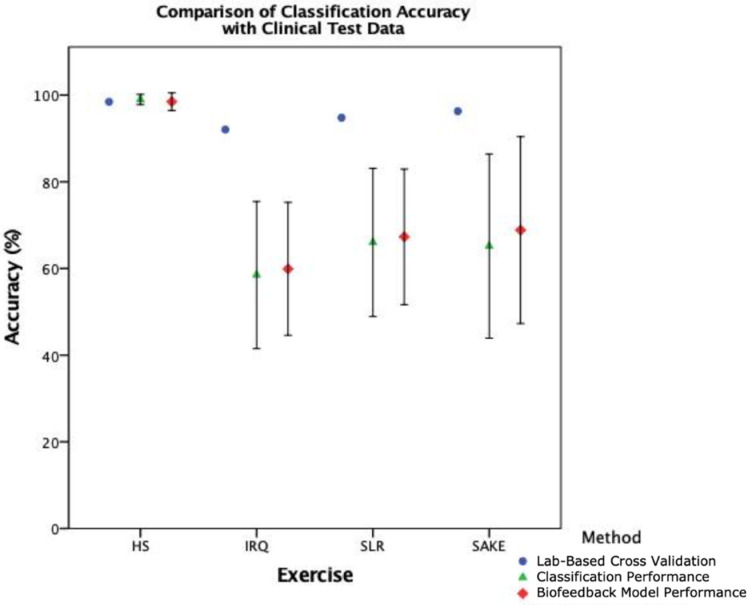
The difference in classification accuracy between the lab-based cross-validation, manually segmented classification performance, and automatically segmented biofeedback model performance when testing with clinical data.

The coordinates generated automatically by the segmentation algorithm were then passed through the classification models in order to test the overall performance of the biofeedback system. Table 7 shows the average classification performance along with 95% CIs from repetitions that were identified by the segmentation model. Mean accuracy in the healthy cohort was greater than 75% across all four exercises with both HS and SAKE continuing to demonstrate 100% accuracy. In the clinical cohort, classification accuracy ranged between 59.90% and 98.49% across the four exercises with algorithmically generated segment coordinates. As illustrated in Figure 8, there was a slight improvement in accuracy in the overall biofeedback model performance of clinical test data compared to the manually segmented classification performance results; however, both were markedly reduced from the lab-based LOSOCV.

**Figure 9:**
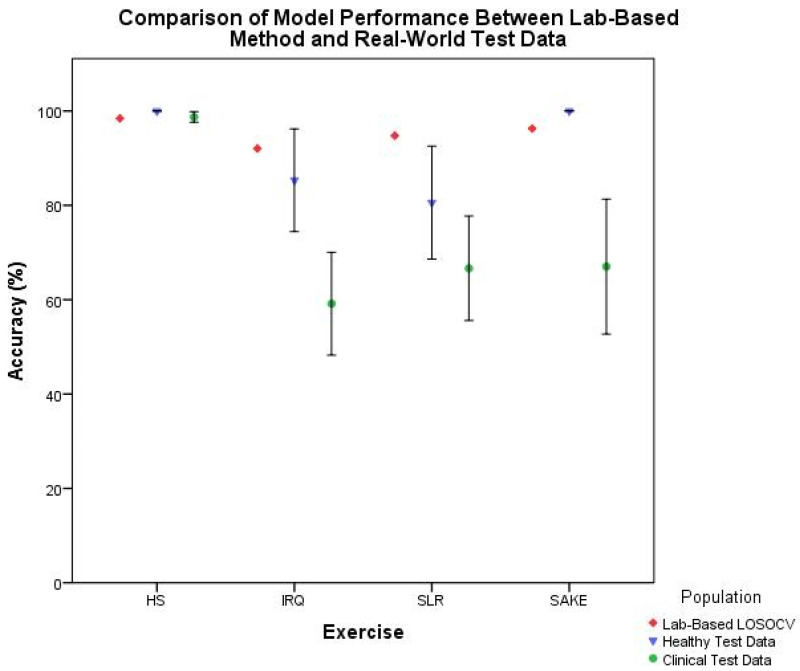
Comparison of overall biofeedback model performance between lab-based cross-validation, and healthy and clinical test data.

Finally, Figure 9 illustrates the difference in combined performance of the ML models between the healthy and clinical population, with the lab-based LOSOCV as a reference. The SAKE demonstrated the largest reduction in mean classification accuracy between the healthy and clinical cohort with a 31.10% reduction, IRQ shows a 26.10% decrease, and SLR a 9.17% loss of accuracy. The HS performance remained largely similar although no sub-optimal examples were included in either test set for this exercise.

## 4. Discussion

This study has highlighted the importance of thorough validation of ML models in IMU based exercise biofeedback systems to ensure acceptable accuracy. The example system evaluated has illustrated the variations in performance across each phase of the process and demonstrates the shortcomings in accuracy of this system in the final clinical deployment, with the overall biofeedback model provided with a balanced clinical test set demonstrating ‘poor’ to ‘moderate’ levels of accuracy. Therefore, this particular model requires further development and refinement prior to wider scale implementation. This study highlights the importance of context when evaluating the performance of rehabilitation biofeedback systems. Making judgements on potential for performance in the real-world setting based on laboratory validation of underlying data models potentially reports an over-optimistic and unrealistic expectation of system accuracy, which in turn has implications for clinical applications.

Making direct comparisons between these results and the literature is difficult due to the lack of external validation that is conducted on ML models [8,17]. The majority of current research outlines LOSOCV and other similar lab-based methods such as k-fold cross-validation [8,10,34,35], rather than the evaluation of ML models with a newly collected and independent test-set. Shany et al. [17] recommended that external validation is the most preferred option in the evaluation of ML models for fall risk prediction, and Figure 9 illustrates the trend in reducing accuracy with external test data in this clinical context. As such cross-validation results should arguably be seen as the best-case scenario rather than actual performance. This study has therefore taken the preferred validation option [17], built on previous work [11,21], and moved past the lab-based validation methods into the real-world setting.

When breaking down the ML components of the system, it is clear that there is a notable reduction in performance in the clinical cohort across both classification (Table 5) and segmentation (Figure 7) when compared to healthy participants. However, interestingly, the slight improvement in overall biofeedback model performance, compared to classification performance in Figure 8, would suggest that the reduction in segmentation accuracy has less of an effect on overall system functionality. This would indicate that the classification component, and the training data provided to the model, is the leading cause for the reduction in accuracy when deploying this system with clinical patients. 

Therefore, this study has highlighted key criteria in the development of exercise classification models, particularly regarding the training data on which the models are built. Firstly, whilst the training data contained data from participants aged between 40 and 80 years of age undergoing lower limb rehabilitation [21], none of the participants were undertaking rehabilitation following TKR or UKR. This could explain the reasons for the reduction in accuracy, as the classification models are being tested against training data which are too heterogenous, and must be closer matched to the population for which they are designed [26]. During data collection using the example system, some participants preferred to complete their exercises on their bed, others on the sofa and some lying on the floor, thus demonstrating the heterogeneity of real-world use. However, the training data were collected in a controlled clinic environment. The presence of thick duvets, soft mattresses, and varying sizes of towels at home can all contribute to variance in the IMU data obtained, and therefore the outputs of the ML models. For pragmatic reasons, most training data sets for exercise biofeedback systems are collected in controlled environments; however, the results of this study would suggest that any ML model must be built on training data from those with the same clinical pathology and collected in the same manner as the target user to demonstrate acceptable levels of accuracy. Equally, it is arguable that given the greater balance between correct and sub-optimal repetitions in the clinical test data, these results are a better reflection of the actual performance of the models and that a balanced test set from healthy participants may have demonstrated a similar reduction in performance [36]. Developers of these systems, and those reviewing their performance, must be cognisant of collecting a balanced test-set, whilst doing their best to avoid including deliberately produced examples of sub-optimal repetitions. Additionally, in this particular approach, further work in refining the classification models is required prior to clinical deployment, including exploring other feature selection approaches and hyperparameter tuning [12].

This binary approach using supervised ML also has implications to both the technical and clinical feasibility of such systems. The need to collate large quantities of training data which contains both raw IMU data and video to label the ground truth is time consuming and requires access to large numbers of participants. There is subjectivity in labelling the exercise technique [37], and the scalability of the method is impacted should it be sought to develop models for additional exercises. Finally, the clinical feasibility of using ML to classify exercise technique can be further questioned, as this method removes the human factors of therapy and the context of each individual patient. For example, it is possible that two patients performing the same exercises with the same clinician will receive markedly different feedback in clinical practice. This is due to the judgement the therapist makes on numerous factors including the rehabilitation timeline, patient’s personality, progress and goals. One patient may be ahead of schedule and moving quickly onto the next exercise, whereas another may be slow and lacking engagement, requiring a different message to be given by the therapist. Whilst in theory it is possible to train a machine to learn these variables, it is not necessarily feasible. As an alternative, during a user evaluation of this example system patients expressed a wish for a greater granularity in the feedback they receive such as joint angle or a quality score [6].

There are a number of limitations to consider when reviewing the results of this study. It is important to highlight that this evaluation took place outside of the custom-built tablet application. This was done to ensure that there were no computational load or software programming issues; however, any such stability concerns must be considered when evaluating a system in its entirety. Secondly, the clinical test set contained multiple data sets from the same participant as they were tested over three different time points. Whilst it was outside the scope of the aims and objectives of this study, further analysis of exercise biofeedback performance within and between participants may be beneficial. Finally, as highlighted previously, the unbalanced test set for the HS exercise means that it is difficult to draw firm conclusions on the functionality of the models for this exercise.

Despite these limitations, this study has highlighted the deterioration in technical functionality of the ML models within an example biofeedback system when deployed with clinical patients compared to healthy controls. The results provide a clear illustration on the importance of not relying on the lab-based validation methods frequently reported in the literature and emphasises that this real-world validation is a crucial step in the development and implementation of IMU-based biofeedback ML systems. 

## 5. Conclusions

The reliance on lab-based validation approaches may be misleading key stakeholders in the IMU-based exercise biofeedback sector. Clinicians should question validation results presented with these cross-validation methods, and researchers should work to demonstrate real-world validity in a newly collected, unseen and balanced test data set. Additionally, conducting this evaluation process will allow developers to assess and understand the factors contributing to ML model performance in order to make modifications. In this example, there was a significant reduction in real-world performance compared to lab-based validation processes, largely due to the heterogeneity of the training set used and variance in the environment patients choose to conduct rehabilitation at home. Finally, the technical and clinical challenges in providing binary exercise biofeedback using supervised ML mean that other options should be explored to support patients in their rehabilitation.

## Figures and Tables

**Table 1:** Description of exercises and the errors assessed within the machine learning models.

Exercise	Description of Exercise	Error Assessed
Heel Slide (HS)	In supine lying, the exercise is performed by flexing the hip and knee to slide the foot closer to the ipsi-lateral hip.	Excessive hip external rotation
Inner Range Quadriceps (IRQ)	In supine lying, a roll is placed under the knee to be exercised. The exercise is performed by contracting the quadriceps muscles to bring the knee from a position of slight flexion into full extension.	Hip flexion (raising knee off the towel)
Straight Leg Raise (SLR)	In supine lying, the exercise is performed by flexing the hip, lifting the leg off the supporting surface while keeping the knee in full extension, raising to a height above the contralateral toes.	Knee flexion (lag)
Seated Active Knee Extension (SAKE)	In sitting with the upper thigh supported on a chair, the exercise is performed by contracting the quadriceps to bring the knee from a position of flexion into full extension.	Lack of full knee extension

**Table 2:** Characteristics of the classification training data.

Exercise	Participants	Exercise Sets	Total Repetitions	Correctly Performed Repetitions	Sub-Optimally Performed Repetitions
HS	36	71	711	350 (49.2%)	361 (50.8%)
IRQ	35	68	679	351 (51.7%)	328 (48.3%)
SLR	37	69	689	370 (53.7%)	319 (46.3%)
SAKE	38	76	754	380 (50.4%)	374 (49.6%)

**Table 3:** Lab-based results following leave-one-subject-out cross-validation.

Exercise	Best Performing Algorithm	Metric (%)		
		Accuracy	Sensitivity	Specificity
HS	Logistic Regression	98.45	99.43	97.51
IRQ	Logistic Regression	92.05	93.73	90.24
SLR	SVM	94.78	96.22	93.10
SAKE	Random Forest	96.29	96.52	96.05

**Table 4:** Data collected to form the test sets.

Cohort	Exercise	Participants	Exercise Sets	Total Repetitions	Correctly Performed Repetitions	Sub-Optimally Performed Repetitions
Healthy	HS	10	10	148	148 (100%)	0 (0%)
	IRQ	10	10	150	150 (100%)	0 (0%)
	SLR	10	10	150	150 (100%)	0 (0%)
	SAKE	10	10	150	150 (100%)	0 (0%)
Clinical	HS	10	23	320	320 (100%)	0 (0%)
	IRQ	10	18	270	203 (75.2%)	67 (24.8%)
	SLR	10	21	297	148 (49.8%)	149 (50.2%)
	SAKE	11	17	241	103 (42.7%)	138 (57.3%)

**Table 5:** Classification performance following manual segmentation of test data.

Cohort	Exercise	Metric (%)								
		Accuracy			Sensitivity			Specificity		
		Mean	(95% CI)		Mean	(95% CI)		Mean	(95% CI)	
			LB	UB		LB	UB		LB	UB
Healthy	HS	100.00	(100.00	100.00)	100.00	(100.00	100.00)	N/A *	N/A *	N/A *
	IRQ	84.67	(67.83	100.00)	84.67	(67.83	100.00)	N/A *	N/A *	N/A *
	SLR	84.67	(68.14	100.00)	84.67	(68.14	100.00)	N/A *	N/A *	N/A *
	SAKE	100.00	(100.00	100.00)	100.00	(100.00	100.00)	N/A *	N/A *	N/A *
Clinical	HS	98.99	(96.46	100.00)	98.99	(96.46	100.00)	N/A *	N/A *	N/A *
	IRQ	58.49	(41.50	75.45)	52.70	(31.93	73.46)	78.17	(51.33	100.00)
	SLR	66.01	(48.93	83.10)	49.14	(20.16	78.13)	81.35	(62.38	100.00)
	SAKE	65.17	(43.92	86.41)	61.12	(22.23	100.00)	68.00	(37.36	98.64)

* due to the unbalanced test set with no sub-optimal repetitions it was not possible to calculate specificity.

**Table 6:** Segmentation model performance with use of the test data.

Cohort	Exercise	Metric (%)								
		Precision			Recall			Accuracy		
		Mean	(95% CI)		Mean	(95% CI)		Mean	(95% CI)	
			LB	UB		LB	UB		LB	UB
Healthy	HS	96.21	(94.03	98.39)	95.56	(92.97	98.15)	92.24	(87.97	96.51)
	IRQ	96.00	(92.67	99.34)	96.00	(92.67	99.34)	92.64	(86.69	98.60)
	SLR	96.23	(94.15	98.32)	93.67	(89.86	97.47)	90.48	(85.64	95.33)
	SAKE	94.33	(90.27	98.39)	94.33	(90.27	98.39)	89.76	(82.62	96.90)
Clinical	HS	86.84	(77.35	96.34)	74.95	(62.87	87.03)	70.64	(58.16	83.12)
	IRQ	79.28	(60.85	97.71)	78.08	(59.96	96.20)	75.03	(57.11	92.94)
	SLR	91.21	(81.19	100.00)	84.48	(71.88	97.08)	81.50	(68.77	94.23)
	SAKE	91.53	(87.85	95.21)	82.05	(74.19	89.92)	77.01	(68.45	85.57)

**Table 7:** Biofeedback model performance: classification results of healthy test data with segments generated automatically.

Cohort	Exercise	Metric (%)								
		Accuracy			Sensitivity			Specificity		
		Mean	(95% CI)		Mean	(95% CI)		Mean	(95% CI)	
			LB	UB		LB	UB		LB	UB
Healthy	HS	100.00	(100.00	100.00)	100.00	(100.00	100.00)	N/A *	N/A *	N/A *
	IRQ	86.00	(68.67	100.00)	86.00	(68.67	100.00)	N/A *	N/A *	N/A *
	SLR	76.47	(56.09	96.86)	76.47	(56.09	96.86)	N/A *	N/A *	N/A *
	SAKE	100.00	(100.00	100.00)	100.00	(100.00	100.00)	N/A *	N/A *	N/A *
Clinical	HS	98.49	(96.46	100.00)	98.49	(96.46	100.00)	N/A *	N/A *	N/A *
	IRQ	59.90	(44.56	75.24)	53.46	(34.35	72.56)	79.23	(58.98	99.49)
	SLR	67.30	(51.66	82.94)	45.34	(19.14	71.55)	87.26	(77.02	97.50)
	SAKE	68.86	(47.29	90.43)	60.48	(18.60	100.00)	74.72	(45.49	100.00)

* due to the unbalanced test set with no sub-optimal repetitions, it was not possible to calculate specificity.

## Data Availability

Date sharing not applicable.

## Supplementary Materials

The following are available online at https://www.mdpi.com/article/10.3390/s21072346/s1, Supplementary File 1: Lab-based results for all classification models developed and tested.
