# Supplementary material for: The Importance of Real-World Validation of Machine Learning Systems in Wearable Exercise Biofeedback Platforms: A Case Study
Source: Sensors (Basel). 2021 Mar 27;21(7):2346. doi: 10.3390/s21072346 (PMC8037109; doi:10.3390/s21072346)
Supplement: Supplementary file 1 [file sensors-21-02346-s001.pdf]

Supplementary File 1:

Algorithm	Metric (%)	Exercise			
		HS	IRQ	SLR	SAKE
Logistic Regression	Accuracy	98.45	92.05	94.63	95.76
	Sensitivity	99.43	93.73	95.95	96.26
	Specificity	97.51	90.24	93.10	95.26
Support Vector Machine with SMO	Accuracy	98.17	89.40	94.78	96.29
	Sensitivity	99.14	90.31	96.22	96.52
	Specificity	97.23	88.41	93.10	96.05
Adaptive Boosting	Accuracy	98.17	90.87	93.90	95.23
	Sensitivity	99.43	90.60	95.95	95.19
	Specificity	96.95	91.16	91.54	95.26
Random Forest	Accuracy	98.03	88.07	90.71	96.29
	Sensitivity	98.86	87.46	92.16	96.52
	Specificity	97.23	88.72	89.03	96.05
J48	Accuracy	96.06	83.80	89.55	95.09
	Sensitivity	96.29	85.19	91.62	95.99
	Specificity	95.84	82.32	87.15	94.21

LOSOCV results for all ML models trained and tested. The best performing algorithm for each exercise is highlighted in green and used within the example system.
